# Task-Irrelevant Novel Sounds have Antithetical Effects on Visual Target Processing in Young and Old Adults

**DOI:** 10.3389/fnagi.2017.00348

**Published:** 2017-10-31

**Authors:** Erich S. Tusch, Nicole C. Feng, Phillip J. Holcomb, Kirk R. Daffner

**Affiliations:** ^1^Laboratory of Healthy Cognitive Aging, Department of Neurology, Brigham and Women’s Hospital/Harvard Medical School, Boston, MA, United States; ^2^NeuroCognition Laboratory, Department of Psychology, Tufts University, Medford, MA, United States

**Keywords:** aging, cross-modal processing, auditory processing, target P3, novelty P3, reorienting negativity

## Abstract

In young adults, primary visual task processing can be either enhanced or disrupted by novel auditory stimuli preceding target events, depending on task demands. Little is known about this phenomenon in older individuals, who, in general, are more susceptible to distraction. In the current study, age-related differences in the electrophysiological effects of task-irrelevant auditory stimuli on visual target processing were examined. Under both low and high primary task loads, the categorization/updating process in response to visual targets preceded by auditory novels, as indexed by the target P3 component, was enhanced in young, but diminished in old adults. In both age groups, the alerting/orienting response to novel auditory stimuli, as measured by the P3a, was smaller under high task load, whereas redirecting attention to the visual task after a novel auditory event, as indexed by the reorienting negativity (RON), tended to be augmented under high task load. Old subjects generated a smaller P3a and RON. We conclude that task irrelevant novel auditory stimuli have the opposite effect on the processing of visual targets in young and old adults. This finding may help explain age-related increases in the disruption of primary task activity by irrelevant, but salient auditory events.

## Introduction

One hypothesized consequence of the aging process is increased susceptibility to distraction by irrelevant stimuli (Friedman et al., 1998; Andrés et al., 2006; Parmentier and Andrés, 2010). In the laboratory setting it has been shown that older individuals demonstrate enhanced early processing of irrelevant auditory stimuli during a visual oddball task (Tusch et al., 2016a), and are more susceptible to distraction when performing working memory (Gazzaley et al., 2008; de Fockert et al., 2009), and reading-with-distraction tasks (Carlson et al., 1995; Li et al., 1998).

Unlike their older counterparts, among young adults, task-irrelevant auditory stimuli have been found to both inhibit and enhance performance on a primary visual task. A theoretical framework for explaining such paradoxical effects of novel auditory events has been presented by SanMiguel et al. (2010a,b). Novel auditory stimuli elicit several different attentional processes, including an alerting response and shift of attention (Parmentier et al., 2010; SanMiguel et al., 2010a,b). Increased alertness/arousal elicited by a novel stimulus has been associated with beneficial effects on the processing of task-relevant events (Fernandez-Duque and Posner, 1997; Schomaker and Meeter, 2014). By contrast, the orienting of attention to a novel sound has been linked to a cost in efficient processing of task-relevant visual stimuli (Schröger and Wolff, 1998; Parmentier et al., 2008). According to SanMiguel et al. (2010a,b), novel sounds are most likely to enhance processing and performance on the primary task when the load is low, the experimental pace is slow, and arousal is limited. In this context, the alerting benefit is greater than the orienting cost, and processing and performance in response to task-relevant stimuli are improved (SanMiguel et al., 2010a,b).

Schomaker and Meeter (2014) link the orienting response and subsequent beneficial effects on primary task performance to locus coeruleus-norepenephrine (LC-NE) function. LC-NE activity likely facilitates task-related decision-making processes and suppresses non-target-related activity (Aston-Jones and Cohen, 2005; Nieuwenhuis et al., 2005). Through this process, unexpected or novel sounds can promote visual processing (Bernstein et al., 1973; Valls-Solé et al., 1995; Hackley and Valle-Inclán, 1998; Wetzel et al., 2012; Schomaker and Meeter, 2014). In contrast, novel sounds are more likely to serve as distractors when task load is high, the experiment is fast-paced, and the subject has an optimal level of arousal. Under these circumstances there is a limited “alerting benefit,” which is outweighed by the “orienting cost,” reflecting the diversion of limited processing resources away from task-pertinent events (SanMiguel et al., 2010a).

The current study utilizes an experiment well-suited for investigating how age and primary visual task load modulate the impact of novel sounds. The primary visual task was administered under low and high load. While low load was consistent across subjects (one target stimulus), high load was individually tailored during a pre-task assessment resulting in a similar level of performance accuracy across participants. This approach aimed to reduce the confound between age- and performance-related effects on indices of underlying information processing (Riis et al., 2008; Haring et al., 2013; Tusch et al., 2016a).

While much of previous research has focused on behavioral effects of cross-modal task-irrelevant stimuli, the current study examines primary task performance, as well as ERP measures of target processing in the primary modality and distractor processing in the task-irrelevant modality. The target P3 in the task-relevant modality was measured, which indexes the process of categorizing an event (Squires et al., 1973; Kok, 2001) or updating memory after an event has been categorized (Donchin, 1981; Donchin and Coles, 1988; Polich, 1996, 2007). Larger P3 amplitude indicates more cognitive resources committed to stimulus processing (Wickens et al., 1983; Polich, 1996) and has been shown to correlate with task performance in some studies (Walhovd and Fjell, 2003; Tusch et al., 2016b). Of particular interest is the difference in P3 amplitude to visual targets preceded by auditory novels compared to visual targets preceded by repetitive auditory standards or no auditory stimuli.

Difference waves, calculated as the difference between electrophysiologic responses to task-irrelevant novel and standard auditory stimuli, were also examined. Specifically, we were interested in two components derived from difference waves: the novelty P3a and reorienting negativity (RON), in response to task-irrelevant auditory stimuli. There is evidence that the novelty P3a reflects different aspects of attentional processing, including alerting, orienting, and executive control (Escera et al., 1998; Daffner et al., 2003; Barcelo et al., 2006; Berti, 2008; SanMiguel et al., 2010b). The RON, which occurs after the P3a, has been proposed to index the refocusing of attention and resources from the task-irrelevant event back toward the primary visual task (Schröger and Wolff, 1998; Munka and Berti, 2006; Berti, 2008; SanMiguel et al., 2008). ERP indices of distractor and target processing provide an opportunity to not only more fully explain behavioral findings, but also detect processing differences that may have slight or no measurable effect on behavior, e.g., compensatory activity that permits older adults to perform at a level similar to that of young adults (Reuter-Lorenz and Cappell, 2008; Daffner et al., 2011; Alperin et al., 2014).

In the current study, we intend to further investigate the effects of cross-modal task-irrelevant stimuli in young and old subjects. We expect the impact of task-irrelevant novel sounds on primary task performance to be mediated by subject age and task load. Previous studies have shown that on average, older individuals exhibit less efficient and reduced overall information processing capacity than younger adults (Salthouse, 1996; Mattay et al., 2006; Reuter-Lorenz and Cappell, 2008; Daffner et al., 2011). This observation leads to the prediction that when primary visual task load is low, novel auditory stimuli preceding visual targets may have the opposite impact on young compared to old adults, leading to augmented target P3 and improved task performance in young subjects (SanMiguel et al., 2010b), but diminished target P3 and worse task performance in old subjects. In contrast, we anticipate that under a high primary task load that is demanding for both young and old subjects, novel auditory stimuli preceding visual targets will lead to reduced target P3 amplitude and inferior task performance in both age groups.

We also predict that, in response to novel auditory stimuli, old subjects will generate a smaller RON than young subjects, which will be associated with worse performance on the primary visual task, reflecting greater difficulty in reorienting attention back to task-relevant events. Since the presentation of novel sounds has been associated with both facilitation and disruption of visual target processing, it is unclear whether the amplitude of the novelty P3a will be predictive of task performance. If alerting/orienting to task-irrelevant novel auditory stimuli reflects a breakdown of executive control over primary task activities, one might expect an age-related increase in the P3a. The impact of primary task load on the P3a to novel auditory events also is uncertain. Our work (Simon et al., 2016; Tusch et al., 2016a) and that of others (SanMiguel et al., 2010a; Sörqvist and Marsh, 2015; Sörqvist et al., 2016) lead to the prediction that higher primary task difficulty protects against distraction by increasing focal-task engagement, which would result in a smaller novelty P3a under the high load for both age groups. Finding such a result would pose a challenge to the load theory of attention (Lavie et al., 2004; Lavie and De Fockert, 2005), which posits that carrying out a more demanding primary task results in enhanced competition for the pool of limited executive control resources. This leads to increased distraction by task-irrelevant events (de Fockert et al., 2001; Lavie et al., 2004; Lavie and De Fockert, 2005; Rissman et al., 2009; Burnham, 2010; Kelley and Lavie, 2011), which in the current study would be indexed by an increase in the amplitude of the novelty P3a.

Results from ERP studies investigating the impact of deviant stimuli that occur within the same modality as target stimuli lead to predictions similar to those derived from the load theory of attention, but for different reasons. In a series of studies, Polich and colleagues (Comerchero and Polich, 1998, 1999; Katayama and Polich, 1998; Polich, 2007) have shown that the amplitude of the P3a to rare, deviant events is modulated by task demands. Making the discrimination between targets and standards more difficult results in an augmentation of the P3a to deviant events presented in the same modality. These investigators hypothesize that the shifting of attention to infrequent, deviant stimuli, as indexed by the P3a, is of larger magnitude when primary task difficulty is high and requires greater engagement of focal attention (Comerchero and Polich, 1998; Polich, 2007). If a similar mechanism underlies the processing of cross-modal deviant events, one would predict that in the current study, infrequent auditory stimuli would elicit a larger P3a when the primary visual task is more demanding, as occurs under the high load condition.

## Materials and Methods

### Participants

Participants were recruited through community announcements in the Boston metropolitan area, including through the Harvard Cooperative Study on Aging. This study was carried out in accordance with the recommendations of the Partners Healthcare System Human Research Committee with written informed consent from all subjects. All subjects gave written informed consent in accordance with the Declaration of Helsinki. The protocol was approved by the Partners Healthcare System IRB (protocol number 2008p001897). Participants also completed a detailed screening evaluation that included a structured interview to obtain a medical, neurological, and psychiatric history; a formal neurological examination, an audiological evaluation, and a test of visual acuity via Snellen eye chart; and the completion of a neuropsychological test battery and questionnaires surveying mood and activities of daily living.

To be included in this study, participants had to be English-speaking, have ≥12 years of education, have a Mini Mental State Exam (MMSE; Folstein et al., 1975) score ≥26, and have an estimated intelligence quotient (IQ) on the American National Adult Reading Test (AMNART; Ryan and Paolo, 1992) ≥100. Participants were divided into two age groups: 18–32 years (young) and 60–79 years (old). Participants were excluded if they had a history of central nervous system (CNS) diseases or major ongoing psychiatric disorders based on DSM-IV criteria (American Psychiatric Association, 1994), focal abnormalities on neurological examination consistent with a CNS lesion, a history of clinically significant medical diseases, or corrected visual acuity worse than 20/40. Participants were also tested with pure tone audiometry in which hearing thresholds were tested at 250, 500, 1000, 2000 and 4000 Hz, and excluded if they demonstrated the following abnormalities: >40 dB mean loss across frequencies, >20 dB difference between ears at any frequency, or >30 dB difference between the best and worst threshold (Friedman et al., 1998; Tusch et al., 2016a). To prevent inclusion of older individuals who may be suffering from mild cognitive impairment or the very early stages of a dementing illness, participants were excluded if their mean percentile performance relative to age-appropriate norms across selected neuropsychological tests (described below) was in the bottom third (below the 33rd percentile). Participants were paid for their time.

To avoid conflating changes in neural activity that are specifically due to differences in age with those due to differences in cognitive ability or task performance, it is crucial to limit differences between age groups on task performance or cognitive capacity (Daselaar and Cabeza, 2005; Riis et al., 2008; Daffner et al., 2011). Daselaar and Cabeza (2005) argue in favor of grouping participants based on a battery of neuropsychological tasks that are standardized and therefore generalizable. Due to the role of top-down control in inhibition of early stimulus processing, as well as the role of executive capacity (EC) in normal cognitive aging (de Fockert et al., 2001; Gazzaley et al., 2008; Rissman et al., 2009), age groups were matched in terms of EC relative to age-appropriate norms on neuropsychological tests (see methods in Tusch et al., 2016a).

### Experimental Procedure

**Figure 1:**
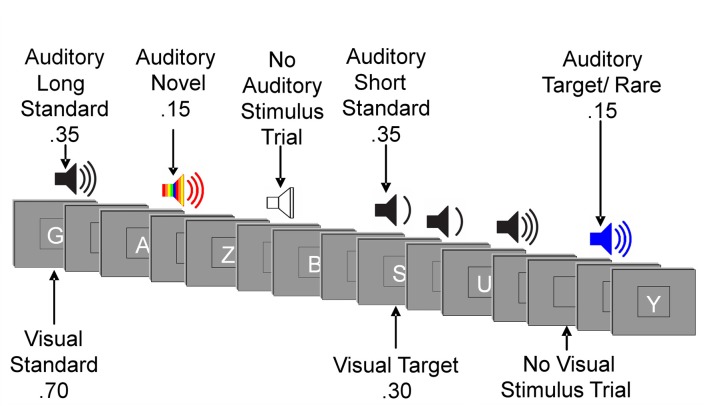
Illustration of an experimental run. Example sequence of visual and auditory stimuli.

The experiment consisted of a forced-choice visual oddball task with irrelevant auditory stimuli. Stimuli were presented using E-Prime software (*E-Prime 2.0*, 2012). Participants were instructed to respond to visual target stimuli and non-target stimuli with opposite mouse clicks (e.g., left click for target stimuli and right click for non-target stimuli). The hand used for the target response was counterbalanced across participants. Participants were instructed to respond to letters and ignore sounds. The order of stimulus presentation varied randomly across blocks within tasks and across tasks. Presentation of letters and sounds did not temporally overlap (Figure 1).

Visual stimuli appeared one at a time within a fixation box that remained on the screen at all times and subtended a visual angle of ~3.5 × 3.5° at the center of a high-resolution computer monitor. Visual stimuli subtended an angle of 2.5° along their longest dimension and were presented for 200 ms. Target letters comprised 30% of visual stimuli. Non-target letters comprised 70% of visual stimuli. Auditory stimuli were presented one at a time with a minimum intensity of 75 dB SPL. Decibel level was adjusted for any participant for whom pure tone audiometry showed a mean hearing loss (across tested frequencies) of 0–40 dBs by increasing (from 75 dB) the intensity of sounds by the mean decibel hearing loss (Friedman et al., 1998; Tusch et al., 2016a). Standard auditory stimuli, comprising 70% of auditory stimuli, were 250 Hz pure tones presented for a duration of either 250 ms (35%) or 125 ms (35%). Rare auditory stimuli, comprising 15% of auditory stimuli, were 500 Hz pure tones of either long (250 ms) or short (125 ms) duration. Short and long rare stimuli were not presented in equal proportion: each comprised 80% or 20% of total rare auditory stimuli, counterbalanced across participants. Novel auditory stimuli were complex, environmentally derived or synthesized sounds presented for a duration of 250 ms, comprising 15% of auditory stimuli. Each novel auditory stimulus in the experiment was unique. Auditory stimuli were presented with 20 ms rise/fall times. The inter-stimulus interval (ISI) between auditory and visual stimuli, and between visual and auditory stimuli varied randomly between 315–665 ms (mean ~490 ms) with 1 ms steps and a rectangular distribution.

In addition to the 800 visual (400) and auditory (400) stimulus trials, there were 100 auditory and 100 visual trials devoid of a stimulus (labeled “no stimulus” trials). For visual stimuli, a no-stimulus trial appeared as a blank presentation box, and the interval between auditory stimuli varied randomly between 830–1530 ms. For auditory stimuli, a no-stimulus trial was a period of silence when an auditory stimulus would normally be presented, and the interval between visual stimuli varied randomly between 880–1580 ms. Of note, we elected not to follow the more common practice of using a fixed temporal interval between auditory and visual events (Parmentier et al., 2010; Wetzel et al., 2012) for two main reasons: increasing variability has greater ecological validity and jittering the interval reduces the influence of overlapping waveforms when measuring ERPs (Woldorff, 1993).

The primary task included 800 stimulus trials divided into eight blocks. As noted, 30% of trials presented visual stimuli (letters) from the target category and 70% of trials presented stimuli from the standard category. Under low task load, one letter was designated as a target. Under the high task load, the number of unique target letters in the target category varied across participants and was determined by an individual’s performance on a titration task. During the titration task, participants were tested on consecutive blocks of the visual task without auditory stimuli. The number of unique letters designated as target stimuli varied across blocks. The number of target letters for which participants scored closest to 80% accuracy (calculated as target hit ratio minus false alarm ratio) was chosen to be used for the high visual task load condition. This procedure was adopted to help ensure that the level of difficulty of the primary visual task was similar across participants from different age groups. Although the number of visual target letters varied across participants from four to nine, the percentage of trials categorized as target events was the same for everyone.

Participants visited the laboratory on three occasions. During the first visit, neuropsychological testing, audiometry, and the visual task load titration procedure were completed. During the remaining two visits, primary visual or auditory experimental tasks, with concurrent ERP recordings, were completed. The auditory forced-choice oddball task was performed during a different session (not reported here) using the same kinds of stimuli as in the visual task. The visual and auditory tasks were scheduled approximately 2 weeks apart from each other to reduce any potential order effects. Each task took approximately 45 min to complete. Task order was counterbalanced across participants. In the current article, only ERP data on visual targets preceded by select auditory stimuli will be presented.

### ERP Recordings

An ActiveTwo electrode cap (Behavioral Brain Sciences Center, Birmingham, UK) was used to hold to the scalp a full array of 128 Ag-AgCl BioSemi (Amsterdam, Netherlands) “active” electrodes whose locations were based on a pre-configured montage. Electrodes were arranged in equidistant concentric circles from 10 to 20 system position Cz. In addition to the 128 electrodes on the scalp, six mini bio-potential electrodes were placed over the left and right mastoid, beneath each eye, and next to the outer canthi of the eyes to check for eye blinks and vertical and horizontal eye movements. EEG activity was digitized at a sampling rate of 512 Hz and filtered offline with a bandwidth of 0.016–100 Hz.

### Data Analysis

Statistical analyses were conducted using R software for statistical computing. Significance was set at *p* < 0.05. Greenhouse-Geisser corrected *p* values were used when factors had more than two levels, and partial eta squared (*η*^2^) was used for estimating effects sizes in repeated measures analyses of variances (ANOVAs). Demographic variables and overall percentile performance on the neuropsychological tests for the groups were compared using one-way ANOVA. E-Prime software was used to generate the behavioral data. A “hit” was defined as a correct response if it occurred between 200–1000 ms after stimulus presentation. Mean reaction time (RT) was measured and ratios of target stimuli correctly responded to (target hits) and stimuli incorrectly identified as targets (false alarms) were calculated. Oddball performance was characterized by the nonparametric discrimination index (e.g., sensitivity) A’ and the A’ composite with RT have been used in studies of cognitive aging (Tusch et al., 2016b; Vermeij et al., 2017). A’ is a behavioral performance variable derived from signal detection theory (Grier, 1971; Hannay, 1986) and ranges from 0.5 (chance level) to 1 (perfect discrimination between targets and non-targets). Composite A’ scores were calculated using A’ and mean RT in response to target stimuli. Composite A’ was used to characterize behavior. As a principled combination of both aspects of task performance (discrimination and RT), the use of composite A’ accounts for speed/accuracy trade-offs in processing and diminishes the influence of strategy effects (McNamara and Scott, 2001). Due to technical issues during data collection, behavioral data recorded by E-Prime are missing for two young participants. However, response encoding in the BioSemi EEG files allowed the ERP data to be analyzed.

EEG data were analyzed using ERPLAB (Lopez-Calderon and Luck, 2014) and EEGLAB (Delorme and Makeig, 2004) toolboxes that operate within the MATLAB framework. Raw EEG data were resampled to 256 Hz and referenced off-line to the algebraic average of the right and left mastoids. EEG signals were filtered using an IIR bandpass filter with a bandwidth of 0.03–40 Hz for young and 0.03–30 Hz for old participants (12 dB/octave roll-off for all). Eye artifacts were removed through an independent component analysis. Individual channels that revealed, upon visual inspection, a consistently different pattern of activity from surrounding channels were corrected with the EEGLAB interpolation function. EEG epochs for visual targets preceded by three types of auditory stimulus trials (standard, novel and no-stimulus) across two task loads (low and high) were averaged separately. The sampling epoch for each trial lasted for 1200 ms, including a 200 ms pre-stimulus period that was used to baseline correct the ERP epochs. Trials were discarded from the analyses if they contained baseline drift or movement artifacts greater than 90 μV. Only trials with correct responses were included in the analyses. Participants were excluded from further analyses if their data were excessively noisy due to frequent contamination by motion artifacts or alpha waves.

**Figure 2:**
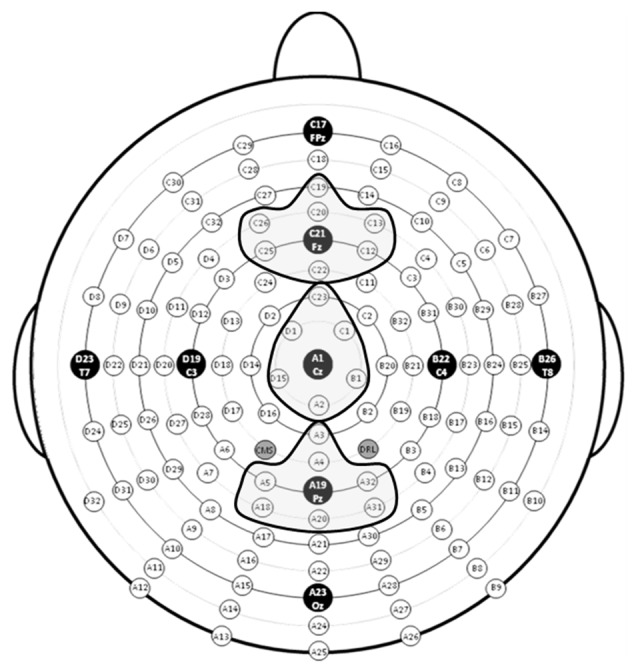
Map of electrode sites with electrode clusters around Fz, Cz and Pz highlighted.

To measure the P3 component in response to targets in the task-relevant visual modality, three regions of interest (ROIs), anterior, central and posterior, were created by averaging clusters of channels centered at midline electrode sites Fz, Cz and Pz (Tusch et al., 2016b; see Figure 2). To measure the novelty P3a and RON in response to stimuli in the task-irrelevant auditory modality, difference waves (ERP responses to novel stimuli minus to standard stimuli) were computed at the Fz ROI.

## Results

### Participants

Table 1 summarizes participant characteristics, including demographic information and neuropsychological test performance for each age group. Twenty-three young and 35 old adults participated in the study. An additional six young and four old participants completed the experiment, but were excluded due to excessively noisy ERP data. The previously mentioned two young participants excluded from behavioral analyses due to missing behavioral data were included in ERP analyses. Only ERPs for target hit trials were analyzed. There were no differences between age groups for EC percentile score based on age-appropriate norms, years of education, estimated IQ (AMNART), or sex. Old subjects had a greater mean hearing loss, *p* < 0.001, and worse corrected visual acuity, *p* < 0.001, than young subjects.

**Table 1:** Demographic and neuropsychological information mean (SD).

	Young	Old	*p* value
N	23	35	*-*
Sex (F:M)	12:11	22:13	n.s.
Age Range in Years	19–30	60–79	-
Mean Age in Years	22.9 (2.7)	71.4 (5.4)	<0.001
Years of Education	15.2 (1.7)	16.1 (3.1)	n.s.
AMNART IQ	117.6 (6.7)	119.7 (9.3)	n.s.
MMSE Score	29.9 (0.3)	29.5 (1.0)	n.s.
EC Percentile Score	67.8 (15.7)	68.8 (16.4)	n.s.
Mean Hearing Loss	0.1 (0.2)	12.6 (9.4)	<0.001
Visual Acuity (corrected)	1.0 (0.2)	0.8 (0.2)	<0.001

*AMNART, American version of the National Adult Reading Test; MMSE, Mini Mental State Exam; Mean Hearing Loss: Average difference across six tested frequencies between measured dB level at threshold and 20 dB; Visual Acuity (corrected): visual acuity of subjects with corrective lenses if applicable; 20/20 = 1.0; 20/25 = 0.8*.

### Task Performance

Since accuracy was used as the key metric during the titration procedure, we report on accuracy results here (see Table 2). Mean RT results are incorporated in the A’ composite scores, reported below. For accuracy, there was an effect of load, *F*_(1,54)_ = 48.12, *p* < 0.001, *η*^2^ = 0.45, but not age group. Performance was superior under low load than under high load. There was no difference in accuracy between age groups under high load (*p* = 0.46). The number of visual targets presented under high task load was greater for young than for the old group (*p* < 0.001).

**Table 2:** Behavioral performance at low and high task load mean (SD).

	Low task load		High task load		
Age group	RT (ms)	Accuracy	Number of visual targets	RT (ms)	Accuracy
Young	489 (47)	0.83 (0.12)	7.61 (1.3)	602 (62)	0.78 (0.11)
Old	585 (49)	0.91 (0.08)	6.40 (1.2)	709 (48)	0.75 (0.15)

**Figure 3:**
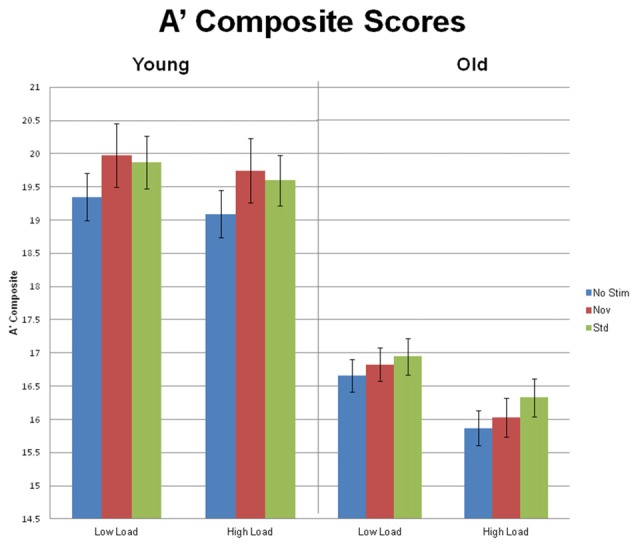
Mean (±SEM) A’ Composite scores for Young and Old subjects at low and high task load, in response to targets preceded by no auditory stimulus, standard and novel auditory stimuli.

Primary visual task performance, measured by A’ composite, was calculated for targets preceded by standard, novel and no-stimulus auditory trials, during both low and high task load. Additional A’ composite scores were calculated by collapsing across the three preceding stimulus types and two loads. Behavior was analyzed via a 2 task load (low and high) × 3 preceding stimulus types (auditory standard, novel and no-stimulus) × 2 age group (young and old) repeated measures ANOVA. Figure 3 summarizes the behavioral results. All three factors demonstrated main effects. There was an effect of task load, *F*_(1,54)_ = 43.79, *p* < 0.001, *η*^2^ = 0.45, such that performance was better during low load than during high. There was also an effect of preceding auditory stimulus type, *F*_(2,108)_ = 4.34, *p* = 0.018, *η*^2^ = 0.07: performance in response to visual targets preceded by no auditory stimulus (A’ composite = 17.74) was inferior to performance in response to targets preceded by standard (A’ composite = 18.19) or novel auditory stimuli (A’ composite = 18.15), with no difference between the latter two. Lastly, there was an effect of age group, *F*_(1,54)_ = 51.25, *p* < 0.001, *η*^2^ = 0.49, such that young subjects outperformed the older age group.

There was an interaction between task load and age group, *F*_(1,54)_ = 10.29, *p* < 0.001, *η*^2^ = 0.16, because the magnitude of the load effect was larger for the old group, *F*_(1,34)_ = 59.55, *p* < 0.001, *η*^2^ = 0.64, than for the young group, *F*_(1,20)_ = 5.39, *p* = 0.03, *η*^2^ = 0.21. Although the interaction between age group and preceding auditory stimulus type did not reach significance, *F*_(2,108)_ = 2.20, *p* = 0.118, we were interested in conducting an exploratory analysis to look for any suggestion that the pattern of behavioral response to targets preceded by different stimulus types may vary within each age group. For young subjects, the effect of preceding stimulus, *F*_(2,40)_ = 4.54, *p* = 0.018, *η*^2^ = 0.19, was due to better performance in response to targets preceded by standards or novels than targets preceded by no stimulus (*p*s < 0.03), with no difference between the former two. In contrast, for old subjects, the effect of preceding stimulus type, *F*_(2,68)_ = 4.72, *p* = 0.015, *η*^2^ = 0.12, was due to better performance in response to targets preceded by standards than targets preceded by no stimulus (*p* = 0.003). A’ composite scores did not differ between targets preceded by novels and targets preceded by no stimulus (*p* = 0.139). The difference between age groups in the impact of novel stimuli vs. no stimulus preceding targets was supported by a significant 2 group × 2 stimulus type (novels vs. no stimulus) interaction, *F*_(1,54)_ = 4.52, *p* = 0.038, *η*^2^ = 0.08.

### Electrophysiology

#### P3 to Visual Target Stimuli

**Figure 4:**
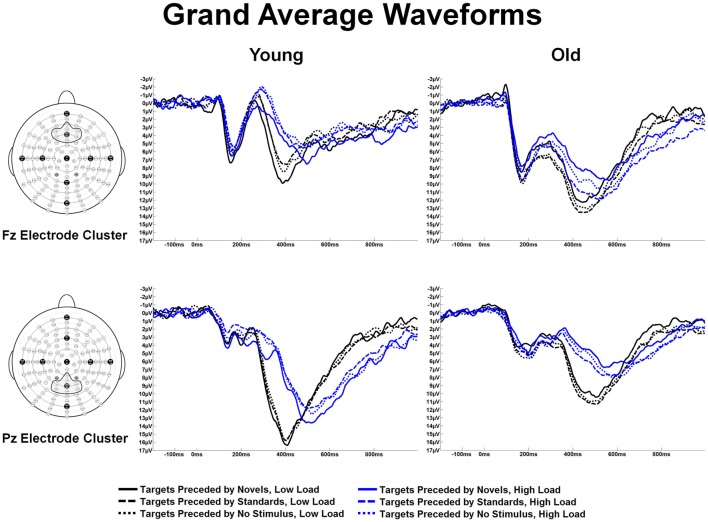
Grand average waveforms at the Fz and Pz electrode clusters for Young and Old subjects at low and high task load, in response to targets preceded by no auditory stimulus, standard and novel auditory stimuli.

Figure 4 illustrates the grand average waveforms in response to target stimuli preceded by the different auditory stimulus types across age groups, ROIs and task load. Local positive peak latencies of the P3 were measured between 350–650 ms for targets preceded by auditory standards, novels, and no-stimulus trials. P3 latency measures were analyzed in a 2 load × 3 ROI × 3 preceding stimulus × 2 age group ANOVA. Of note, there was an effect of load, *F*_(1,56)_ = 68.60, *p* < 0.001, *η*^2^ = 0.55 (shorter latencies under low than high load) and an effect of ROI, *F*_(2,112)_ = 11.30, *p* < 0.001, *η*^2^ = 0.17 (P3 latencies were shorter at the Fz ROI and Cz ROI than the Pz ROI). There was an interaction between age and ROI, *F*_(2,112)_ = 12.48, *p* < 0.001, *η*^2^ = 0.18. This interaction was due to an age group difference at the Pz ROI, *F*_(1,56)_ = 16.33, *p* < 0.001, *η*^2^ = 0.23 (young subjects had shorter P3 latencies than old subjects), but not at the other two ROIs.

Marginal means derived from the significant results observed in the P3 latency ANOVA were used to anchor 150 ms windows for measuring mean amplitude of the P3 to target stimuli. Target P3 amplitude was analyzed via 2 task load × 3 preceding auditory stimulus × 3 ROI × 2 age group ANOVA. There was an effect of ROI, *F*_(2,112)_ = 16.41, *p* < 0.001, *η*^2^ = 0.23, and task load, *F*_(1,56)_ = 27.56, *p* < 0.001, *η*^2^ = 0.33, but not of age (*p* > 0.9) or preceding stimulus type (*p* > 0.5). The ROI effect was due to the largest amplitude being at the Pz ROI, followed by the Cz ROI, and then Fz ROI (*p*s ≤ 0.001). The ROI effect differed across age groups, (ROI × age group interaction) *F*_(2,112)_ = 74.25, *p* < 0.001, *η*^2^ = 0.57, which indicated differing spatial distributions of the target P3. Young subjects demonstrated maximal amplitude at the Pz ROI (effect of ROI, *F*_(2,44)_ = 45.39, *p* < 0.001, *η*^2^ = 0.67) whereas old subjects demonstrated maximal amplitude at the Fz ROI (effect of ROI, *F*_(2,68)_ = 18.80, *p* < 0.001, *η*^2^ = 0.36). An alternative way to characterize this interaction is that young subjects generated a larger P3 amplitude than old subjects at the Pz ROI, *F*_(1,56)_ = 13.36, *p* < 0.001, *η*^2^ = 0.19, whereas old subjects generated a larger P3 amplitude than young subjects at the Fz ROI, *F*_(1,56)_ = 16.83 *p* < 0.001, *η*^2^ = 0.23. Note that P3 responses around Pz are most commonly interpreted as reflecting a greater contribution of P3b activity, while responses around Fz reflect a greater contribution of P3a activity (Knight, 1997; Polich, 2007; Alperin et al., 2014).

The effect of task load indicated that P3 amplitude was larger under low task load than under high task load. This effect was not modified by preceding stimulus type, *p* > 0.7, or age group, *p* > 0.2. An interaction between ROI and load, *F*_(2,112)_ = 13.80, *p* < 0.001, *η*^2^ = 0.20, was present because the P3 was more anteriorly distributed under high than low load. This interaction was not further modified by age group, *p* > 0.3.

**Figure 5:**
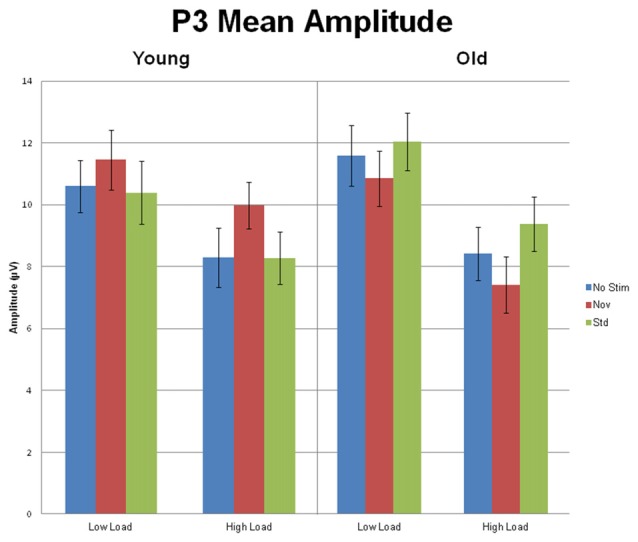
Mean (SEM) amplitude of the P3 (collapsed across the threeelectrode clusters) for Young and Old subjects at low and high task load, in response to targets preceded by no auditory stimulus, standard and novel auditory stimuli.

**Figure 6:**
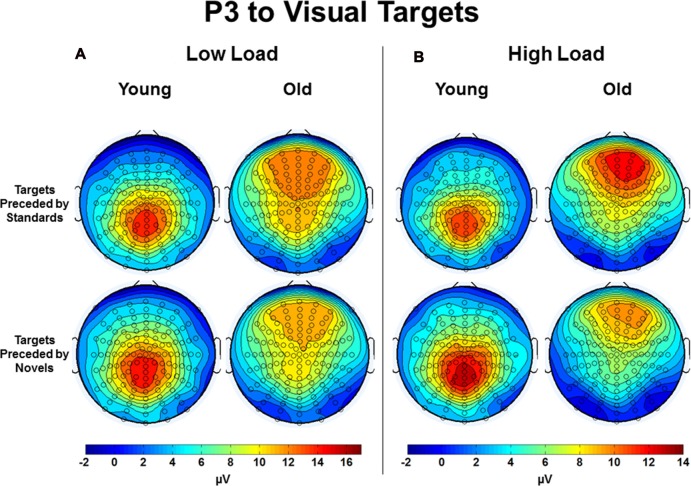
Topographic voltage maps of the mean amplitude of the P3 to visual targets preceded by auditory standards and visual targets preceded by auditory novels in Young and Old subjects under **(A)** low task load and **(B)** high task load. Note the voltage scales differ between low load and high load.

Of particular interest to the goals of this investigation was an interaction between preceding auditory stimulus type and age group, *F*_(2,112)_ = 16.44, *p* < 0.001, *η*^2^ = 0.23 (see Figure 5 for a bar graph summarizing the data). For young subjects, the effect of preceding auditory stimulus type, *F*_(2,44)_ = 5.80, *p* = 0.01, *η*^2^ = 0.21, was due to the P3 amplitude in response to targets preceded by novel auditory stimuli being larger than to targets preceded by standards or no stimulus; P3 responses to targets preceded by auditory standards or no auditory stimulus did not differ. In contrast, for old subjects, the effect of preceding auditory stimulus type, *F*_(2,68)_ = 12.57, *p* < 0.001, *η*^2^ = 0.27, was due to the P3 amplitude in response to targets preceded by standard auditory stimuli being larger than targets preceded by both novel and no stimuli. For illustrative purposes, Figure 6 presents the topographic voltage maps of the mean amplitude of P3 to visual targets preceded by auditory standards and visual targets preceded by auditory novels. The interaction between preceding stimulus type and age group was modified by ROI, *p* = 0.013, due to previously described age-related differences in spatial distribution of the P3 component. The interaction between age group, stimulus type, and load was not significant, *p* > 0.3. However, since we had hypothesized that young subjects would exhibit the opposite pattern of P3 response to targets preceded by novel stimuli under low and high load, whereas old subjects would demonstrate the same pattern, we also examined the interaction between preceding stimulus type and load for each age group separately, and found that neither was significant: young subjects, *p* > 0.5; old subjects, *p* > 0.4.

#### Novelty P3a and RON to Task-Irrelevant Auditory Stimuli

**Figure 7:**
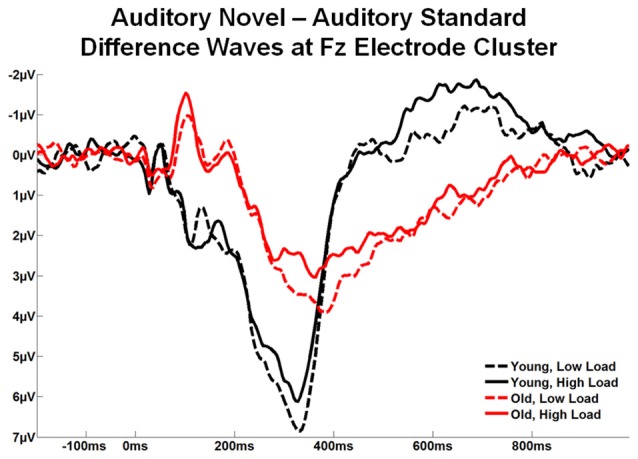
Grand average novel minus standard difference waves at the Fz electrode cluster for Young and Old subjects at low and high task load.

Figure 7 illustrates the difference waves (auditory novel stimuli − auditory standard stimuli) at the Fz ROI across the two age groups and two loads. Local peak latency was measured at Fz ROI for the novelty P3a between 250–500 ms, and for the RON between 500–800 ms. Analyzed by 2 task load × 2 age group ANOVAs, both components exhibited a difference in peak latency between age groups, significant for P3a, *F*_(1,56)_ = 12.26, *p* < 0.001, *η*^2^ = 0.18 and a trend for RON, *F*_(1,56)_ = 2.94, *p* = 0.092, *η*^2^ = 0.05, with the latencies being shorter for young than old subjects. There was no effect of load and no interaction between age group and load.

**Figure 8:**
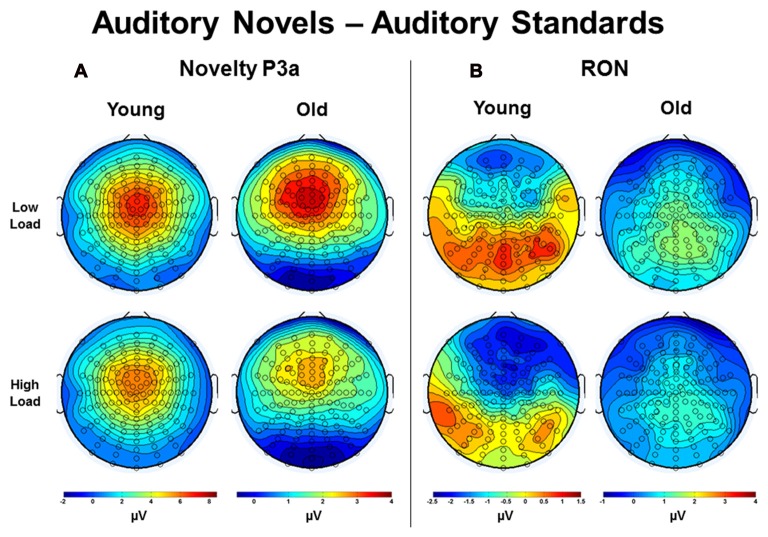
Topographic voltage difference maps (auditory novel stimuli minus auditory standard stimuli) in Young and Old subjects under low and high task load for the **(A)** Novelty P3a and **(B)** reorienting negativity (RON). Note that the voltage scales differ across subject groups.

Estimated marginal means for different age groups were used to anchor 100 ms mean amplitude measurement windows for both difference wave components. Figure 8 illustrates topographic voltage difference maps (auditory novel stimuli − auditory standard stimuli) for the novelty P3a and RON. A task load × age group ANOVA was run for both the novelty P3a and RON novel-standard difference wave amplitude measurements (henceforth referred to as novelty P3a and RON respectively). The effect of task load was significant for the novelty P3a amplitude, *F*_(1,56)_ = 5.80, *p* = 0.019, *η*^2^ = 0.09, and there was a strong trend towards significance for the RON, *F*_(1,56)_ = 3.66, *p* = 0.061, *η*^2^ = 0.06. Crucially, the amplitude difference between loads moved in opposite directions for the two components (novelty P3a: low load > high load; RON: low load < high load). Additionally, the novelty P3a and RON difference wave amplitudes did not correlate with each other (low load, *p* > 0.1; high load, *p* > 0.5, averaged across loads, *p* > 0.6). The findings of opposite load effects and a lack of correlation between novelty P3a and RON components indicate that they did not measure the same underlying process at overlapping temporal intervals.

Both the novelty P3a and RON difference wave amplitude demonstrated a main effect of age group, novelty P3a: *F*_(1,56)_ = 9.84, *p* = 0.003, *η*^2^ = 0.15; RON: *F*_(1,56)_ = 24.70, *p* < 0.001, *η*^2^ = 0.31. Unlike the effect of task load, which indicated different patterns for the novelty P3a and RON difference waves, the effect of age group demonstrated the same pattern for each component: amplitude was greater for young subjects than for old subjects. Of note, old subjects appeared to generate a relatively weak RON. No interaction between age group and task load was observed for either the novelty P3a or RON difference waves (*p*s > 0.4).

All of the ERP analyses for the target P3, novelty P3a and RON were re-run after removing the two young subjects who lacked behavioral data, and demonstrated that the pattern of results remained the same and all analyses that were significant remained so.

#### Electrophysiological/Behavioral Relationships

Because we were particularly interested in understanding the impact of task-irrelevant novel sounds on visual target processing, we examined the relationships between the novelty P3a difference wave, RON difference wave, P3b to visual targets preceded by auditory novels, and A’ composite associated with visual targets preceded by auditory novels. Note that target P3b was measured at the Pz ROI (Knight, 1997; Polich, 2007; Alperin et al., 2014). For simplicity, we highlight the data collapsed across low and high loads. However, with one exception noted, the pattern of relationships observed was similar when analyzing low load or high load alone.

The novelty P3a difference wave amplitude did not correlate with RON difference wave amplitude (*p* > 0.5) or A’ composite (*p* > 0.1), but weakly correlated with target P3b to targets preceded by auditory novels (*r* = 0.38, *p* = 0.003). Unlike the other components, there was no reliable correlation under low or high load alone. The amplitude of the RON difference wave inversely correlated with the size of the target P3b (*r* = −0.36, *p* = 0.005): the larger the RON, the larger the P3b. The amplitude of the RON difference wave also inversely correlated with the A’ composite (*r* = −0.52, *p* < 0.001): the larger the RON, the better the performance. This remained significant after accounting for target P3b amplitude (*r* = −0.45, *p* < 0.001). Target P3b amplitude also correlated with A’ composite (*r* = 0.48, *p* < 0.001), which remained significant after controlling for the size of the RON (*r* = 0.3, *p* = 0.026). In summary, the novelty P3a difference wave had a limited relationship with the RON difference wave, P3b, and behavioral performance. A more robust process of reorienting attention to the primary visual task, as indexed by the RON difference wave, was associated with more robust categorization/updating of target stimuli, as measured by the P3b. Processing indexed by the RON difference wave and P3b both correlated with performance in response to visual targets preceded by auditory novels, as measured by the A’ composite score.

## Discussion

The most critical finding of the study is that when task-irrelevant novel auditory stimuli precede visual stimuli, young and old subjects demonstrate opposite electrophysiological effects on target processing. Consistent with the work of SanMiguel et al. (2010a,b), novel sounds were associated with enhancement of the target categorization/updating process in young subjects, as measured by increased P3 amplitude. In contrast, old adults demonstrated a disruption of this process, as indexed by a reduction in P3 amplitude. These results may help to explain age-related differences in susceptibility to distraction by salient cross-modal, task-irrelevant auditory stimuli (Friedman et al., 1998; Townsend et al., 2006; Horváth et al., 2009; Tusch et al., 2016a).

We had predicted that under high task load, novel auditory stimuli would undermine primary target processing in young subjects. According to the framework proposed (SanMiguel et al., 2010a), under very demanding task conditions, the “alerting benefit” in response to novel sounds is small and overshadowed by the “orienting cost” that diverts capacity-limited resources from the target categorization/updating process. However, we found that the pattern of P3 response to targets preceded by different stimulus types was, in fact, not modulated by task load. It remains unclear whether this result poses a real challenge to the proposed theory. In the current study, the demands of the high task load were individually titrated to achieve ~80% accuracy. It is plausible that this level of difficulty was not sufficient to cause the impact of novel events to shift from enhancement to interference of visual target processing. To help settle this issue, future studies should include even more demanding task loads.

If, as has been proposed, the degree of alerting/orienting activity elicited by novel sounds, as indexed by the P3a difference wave, plays a decisive role in subsequent target processing, one might expect that across subjects, the P3a difference wave amplitude elicited by novel sounds would predict the P3b amplitude and behavioral performance in response to target stimuli preceded by novel sounds. However, the study demonstrated a rather weak correlation between the P3a difference wave and target P3b, and no relationship between the P3a difference wave and performance. These results raise questions about currently hypothesized mechanisms that link task-irrelevant novel events to the primary target processing that follows (SanMiguel et al., 2010a,b; Schomaker and Meeter, 2014).

Our results indicate an age-related decline in the process of returning attention to the primary task after the presentation of a novel sound, as indexed by a smaller, delayed RON in old than young subjects. This finding is consistent with previous reports that have demonstrated age-associated changes in RON latency and amplitude that suggest a less efficient reorienting mechanism in older adults (Mager et al., 2005; Horváth et al., 2009; Getzmann et al., 2013). Findings from the correlation analyses suggest that the process of reorienting attention to the primary task has a greater influence on target processing, as indexed by the target P3b and visual task performance, than does the alerting/orienting response itself, as measured by the P3a. Despite being task-irrelevant, novel auditory stimuli may paradoxically serve to summon participants, especially young ones, to reinvest resources in the primary task. Actively recommitting attention to the primary task, as measured by a robust RON difference wave, was associated with an enhanced target categorization/updating process. Of note, the magnitude of the reorienting of attention after novel sounds did not correlate with the size of the alerting/orienting response to those task-irrelevant novel sounds, suggesting that these two processes are independent of each other (Munka and Berti, 2006).

Interestingly, in both age groups, the amplitude of the novelty P3a difference wave was smaller under high than low primary task load. This finding is consistent with many (Harmony et al., 2000; Berti and Schröger, 2003; Restuccia et al., 2005; SanMiguel et al., 2008), but not all (SanMiguel et al., 2010b) reports about load-associated effects on the amplitude of the novelty P3a. It also accords with prior work in our laboratory, which demonstrated that the N1 response to task-irrelevant auditory events is diminished when a primary visual task is more demanding (Simon et al., 2016; Tusch et al., 2016a).

Our load-related P3a findings support the hypothesis that higher primary task difficulty results in “protection” from distraction by increasing focal task engagement, which has been described in terms of “steadfastness of attention” (Sörqvist and Marsh, 2015; Sörqvist et al., 2016) or the narrowing of the “attentional spotlight” (SanMiguel et al., 2010a). Such activity is hypothesized to facilitate more vigorous suppression of the processing of irrelevant stimuli. The absence of an interaction between task load and age group suggests that the effect of load is of similar magnitude for young and old adults. The decrease in P3a to cross-modal deviant stimuli in the context of greater primary task difficulty appears to be the opposite of what has been reported in intramodal studies. This research has shown that increasing primary visual task demands leads to a larger P3a response to deviant events (Comerchero and Polich, 1998, 1999; Polich, 2007). It remains to be determined whether the conflicting findings are related to the presentation of cross-modal, rather than intra-modal, deviant stimuli, or are the result of different methods for augmenting task difficulty. In the studies by Comerchero and Polich (1998, 1999); Polich (2007), task demands were increased by making the discrimination between target and standard stimuli more difficult, whereas in the current study tasks demands were a function of WM load. Our results are consistent with the notion that the P3a component is modulated by experimental context and is not simply a response to stimulus deviance: identical kinds of auditory stimuli were used under low and high load conditions but elicited very different P3a amplitudes. We suspect that the load-associated reduction of P3a amplitude is a reflection of fewer resources being directed to deviant auditory events. High primary task WM load seems to increase executive control activity that may constrain the processing of stimuli outside of the focus of attention.

It is noteworthy that the observation of a load-related reduction in the amplitude of the novelty P3a to task-irrelevant events is contrary to the predictions derived from the load theory of attention (de Fockert et al., 2001; Lavie et al., 2004; Lavie and De Fockert, 2005; Kelley and Lavie, 2011). This theory suggests that a more cognitively demanding primary task would be associated with the reduced ability for individuals to actively maintain current processing priorities, leading to increased, not decreased, processing of task-irrelevant events. The load-related impact on the processing of task-irrelevant stimuli in our non-spatial task also differs from that associated with exogenous shifts of spatial attention. Although there is strong evidence that increasing perceptual load of centrally presented visual tasks leads to suppression of either visual or auditory exogenous spatial cueing effects, augmenting WM load does not appear to impact the exogenous orienting of spatial attention to task irrelevant stimuli (Santangelo et al., 2007, 2008; Santangelo and Spence, 2008).

An alternative account for our P3a findings is that under high primary task load, there are fewer processing resources available for orienting to task-irrelevant stimuli (Harmony et al., 2000), resulting in a reduced novelty P3a. Interestingly, this account regarding the processing of cross-modal distractors echoes arguments made by classical perceptual load theory as it applies to intra-modal events (Lavie et al., 2004; Lavie, 2005). Our data for the RON strongly argue against this explanation. If, as this account suggests, the load-related reduction in P3a was due to a paucity of available resources under the high task load, one would expect a comparable decline in the amplitude of the RON. However, the opposite was observed: the size of the RON difference wave was larger under the high than the low load condition, a result similar to the load-related increase in RON reported by SanMiguel et al. (2008). Several factors may contribute to the finding of an increased RON. First, under the high load condition, more resources are likely needed to reorient attention in order to manage greater task demands. Some investigators have suggested that refocusing attention onto the task-relevant contents of WM is one aspect of the RON (Munka and Berti, 2006; SanMiguel et al., 2008), which would be substantially larger under the high than low load condition. Finally, consistent with the notion that higher task load elicits greater steadfastness of attention, a brief perturbation of focus in response to novel sounds may lead to an intensification of effort to get back on track. It is important to point out that the magnitude of the load effect for the RON did not differ across age groups, indicating that old subjects preserved the tendency to allocate additional resources to reorienting attention in the face of more demanding primary task conditions.

Behaviorally, the titration procedure employed was successful, generating an average accuracy rate under the high load of close to 80%, which did not differ between age groups. To accomplish this outcome, on average, young subjects needed to be presented with a greater number of target letters than old subjects. Despite the success of the titration procedure, young subjects performed better than old subjects, as measured by A’ composite scores, which take into account both accuracy and speed of processing, the latter of which is invariably reduced in older adults (Salthouse, 1996). As expected, performance was worse under the high load condition, with the magnitude of this effect being larger for old than young subjects (Mattay et al., 2006; Nagel et al., 2009).

There was limited support for our behavioral predictions regarding the impact of preceding auditory stimulus type on target response. For young subjects, both standard and novel auditory stimuli appeared to boost performance compared to when targets were preceded by no stimulus, suggesting that preceding auditory stimuli likely served more as an alerting/warning signal than as a distraction (Fernandez-Duque and Posner, 1997; SanMiguel et al., 2010a; Schomaker and Meeter, 2014). However, no additional behavioral advantage was observed for targets preceded by novel auditory stimuli than for those preceded by standards.

This unanticipated finding may be related to our particular experimental design that differed from studies commonly reported in the literature in which all visual events are preceded at a predetermined interval by auditory stimuli that are either standard or deviant/novel. Because we were interested in determining the cost or benefit of task-irrelevant auditory events on the visual targets that follow, we elected to include trials in which there was no preceding auditory stimulus (Posner, 1978). Mixing visual trials that had no preceding auditory stimulus with trials that had preceding auditory stimuli and making the timing of stimulus presentations variable may have altered the behavioral impact of novel events (Parmentier et al., 2010; Wetzel et al., 2012). Future studies should compare blocks with and without auditory distractors, and with and without fixed temporal intervals between auditory and visual events. Also, contrary to expectation, the pattern of behavioral responses did not change across task load, which, as discussed earlier, may have been due to an insufficiently demanding high load condition.

Results from our exploratory analysis examining whether the pattern of behavioral responses to targets preceded by different stimulus types may vary within each age group raises the possibility that in contrast to their younger counterparts, for old adults, only targets preceded by auditory standards resulted in reliably improved performance relative to targets preceded by no stimulus. There was no performance difference between targets preceded by novels and targets preceded by no stimulus, perhaps suggesting that alerting benefits elicited by novel stimuli were largely counteracted by distraction deficits. However, since the overall interaction between group and preceding stimulus type was not reliable, the interpretation of a differential pattern of behavioral responses between old and young subjects should be viewed as hypothetical, which requires a larger study with greater statistical power to confirm or invalidate it. It remains to be determined why the pattern of behavioral response to targets preceded by various auditory stimulus types differed from the pattern of electrophysiological responses. Dissociations between behavioral and ERP results observed in studies examining the impact of task irrelevant sounds on visual processing are not uncommon (Yago et al., 2001; Polo et al., 2003; Gumenyuk et al., 2004; Yucel et al., 2005; Munka and Berti, 2006; van Mourik et al., 2007). In the current study, there may have been sufficient time between the initial evaluative process (indexed by the target P3) and the behavioral response (indexed by the button press) to have modified the final behavioral output.

In summary, task irrelevant novel auditory stimuli have antithetical effects on the processing of visual targets in young compared to old adults, as indexed by the target P3. These findings may help account for age-related increases in the disruption of pertinent visual processing by salient but task-irrelevant auditory events. In both age groups, intensifying attentional focus on the primary visual task by augmenting load was associated with a reduction in the alerting/orienting response to novel sounds, as measured by the novelty P3a difference wave, and an augmentation of the process of returning attention to the primary task after a novel event, as indexed by the RON difference wave. Finally, the reorienting of attention after the occurrence of a novel sound may be more closely linked to behavioral performance than the alerting/orienting process that precedes it.

## Author Contributions

EST analyzed the data, wrote the initial draft and edited the manuscript. NCF analyzed the data, prepared figures and edited the manuscript. PJH designed the study and edited the manuscript. KRD designed the study, analyzed the data and edited the manuscript.

## Conflict of Interest Statement

The authors declare that the research was conducted in the absence of any commercial or financial relationships that could be construed as a potential conflict of interest.
